# Diet-Dependent Chemical Profiling and Bioactivity of *Otala tingitana* Mucus: Antibacterial Activity, Antioxidant Capacity, and *In Vivo* Wound-Healing Effects

**DOI:** 10.3390/molecules31091499

**Published:** 2026-04-30

**Authors:** Abdelmajid El Khayari, Abdulrahman Mohammed Alhudhaibi, Elhabib Rour, Aziz Bouymajane, Tarek H. Taha, Fouzia Rhazi Filali, Emad M. Abdallah, Abdelaziz Ed-Dra

**Affiliations:** 1 Research Team: Cell Signaling, Department of Biology, Faculty of Sciences, Moulay Ismail University, Meknes 50050, Morocco; elhabib.rour@yahoo.fr; 2 Department of Biology, College of Science, Imam Mohammad Ibn Saud Islamic University (IMSIU), Riyadh 11564, Saudi Arabia; amalhudhaibi@imamu.edu.sa (A.M.A.); thali@imamu.edu.sa (T.H.T.); 3 Bio-Resources, Environment and Health Team, Faculty of Sciences and Techniques of Errachidia, Moulay Ismail University, Errachidia 52000, Morocco; azizbouymajane.01@gmail.com; 4 Laboratory of Chemistry-Biology Applied to the Environment, Faculty of Sciences, Moulay Ismail University, Meknes 50050, Morocco; fouzia.filali@yahoo.fr; 5 Department of Biology, College of Science, Qassim University, Buraydah 52571, Saudi Arabia; 140208@qu.edu.sa; 6 Laboratory of Engineering and Applied Technologies, Higher School of Technology, Sultan Moulay Slimane University, Beni Mellal 23000, Morocco

**Keywords:** *Otala tingitana* mucus, antibacterial activity, anti-inflammatory properties, antioxidant capacity, wound healing, animal-derived therapeutics

## Abstract

Snail mucus is increasingly investigated as a biologically compatible source of multifunctional biomolecules for pharmaceutical and dermatological use. However, the chemical profile and biological activities of mucus from the Moroccan endemic terrestrial snail *Otala tingitana* remain poorly characterized. In addition, the influence of heliciculture diet on the composition and functional properties of the mucus remains unclear. Here, *O. tingitana* was reared for 140 days under controlled conditions and fed a basal flour diet or the same diet supplemented with 3% *Rosmarinus officinalis*, *Origanum compactum*, or *Thymus zygis* subsp. *zygis*. Mucus from wild snails was included for comparison. Mucus samples were chemically profiled by GC–MS and evaluated for antibacterial activity, antioxidant capacity, wound-healing efficacy in mice, and histological anti-inflammatory effects, and evaluated semi-quantitatively based on the degree of inflammatory cell infiltration. GC–MS identified 13 compounds and demonstrated clear diet-dependent shifts in dominant components. All mucus samples exhibited broad-spectrum antibacterial activity against *Staphylococcus aureus*, *Listeria monocytogenes*, *Escherichia coli*, and *Salmonella* Typhimurium (inhibition zones 10.31–14.30 mm; MIC 120–240 µg/mL), with predominantly bactericidal profiles (MBC/MIC < 4) and significantly enhanced activity in plant-supplemented groups *(p* < 0.05). Antioxidant performance improved markedly with medicinal-plant supplementation, reaching low IC_50_ values (best ≈ 1.18 mg/mL) compared with basal-diet mucus. *In vivo*, topical application accelerated wound closure, achieving complete healing in <21 days, versus 28 days in untreated controls. In addition, histological assessment showed faster resolution of inflammatory cell infiltration in treated groups. Collectively, these findings provide the first integrated evidence that *O. tingitana* mucus possesses antibacterial, antioxidant, wound-healing, and anti-inflammatory activities, and that heliciculture diet is a practical lever to optimize its bioactive profile. Further studies should prioritize standardized manufacturing, contaminant control, and safety/toxicology assessment before translational development.

## 1. Introduction

Rising demand for multifunctional biomaterials with antimicrobial, antioxidant, and tissue-repair capacity has intensified interest in natural biosecretions, particularly given the ongoing challenges of chronic wounds, resistant infections, and delayed tissue regeneration [1]. Although synthetic drugs remain central to modern pharmacotherapy, their use can be constrained by adverse effects ranging from mild gastrointestinal disturbances to clinically significant hepatotoxic, nephrotoxic, or immunological complications [2,3]. In addition, the rapid emergence of antimicrobial resistance has significantly compromised the effectiveness of widely used antibiotics, posing a serious global health threat [4]. Moreover, synthetic drugs are frequently designed to target single pathways, which may be insufficient for managing complex, multifactorial diseases such as cancer, metabolic disorders, and chronic inflammatory conditions [5]. These challenges have stimulated growing interest in natural products as alternative or complementary therapeutic agents.

Natural products derived from plants and animals have been considered a vital source of bioactive compounds used in cosmetics, therapeutics, and for nutritional applications [6]. Their bioavailability and compatibility with human structure have made them preferred substitutes or complements for drugs and synthetic chemicals’ precursor used in the medical and pharmaceutical fields [7]. Snails have attracted scientific and industrial interest because they produce diverse natural compounds. Among these, gastropod mucus has emerged as a biologically active secretion relevant to dermatological, antimicrobial, and regenerative research [8]. This hydrated matrix and small metabolites contribute to lubrication, hydration, microbial defense, and tissue protection [9,10,11]. Snails are rich with bioactive compounds, including collagen, allantoin, hyaluronic acid, glycolic acid, and elastin, that have long been utilized to treat inflammatory and dermatological issues [11,12,13]. Where its application in pharmacology, medicine, and cosmetics has increased in the last years [8].

Snail mucus is a viscous, nutrient-rich secretion, produced by snails. Its chemical composition showed high distribution of volatile compounds among esters, alcohols, aldehydes, ketones, and sulfur types. In recent studies, chemical composition of mucus from *Helix aspersa* Müller and *Eremina desertorum* showed their richness in biomolecules [14,15]. This richness provides snail mucus with various pharmaceutical properties. In this context, previous studies have demonstrated the effectiveness of snail mucus in treating burns, lesions, and wrinkles cases, among other therapeutic applications, due to its antimicrobial, antioxidant, anti-inflammatory, regenerative, and anticancer properties [8,11,15,16].

*Otala tingitana* snail, is a Moroccan endemic terrestrial invertebrate belonging to the phylum Mollusca, family Helicidae, the genus *Otala*. This snail species had an optimal growth and reproduction under a temperature of 20 °C and a high relative humidity of 80% [17]. In our previous studies, we were able to domesticate and proceed to sustainable heliciculture of *O. tingitana* under controlled environments by using plant-based feed supplements [18], which opens perspectives in high valorization of its products, either in human alimentation or by exploring its pharmaceutical and medical properties.

Medicinal plants are rich in bioactive compounds with proven pharmaceutical and medical properties, including antimicrobial, antioxidant, anti-inflammatory properties, among others [19,20,21]. In fact, the inclusion of medicinal plants as feed supplements in heliciculture practices has enhanced growth, quality, and reproduction of snail species [18,22,23]. In this perspective, we hypothesize that the inclusion of plants as feed supplements during heliciculture can enhance the pharmaceutical properties of snail mucus. Through this synergy, we can enhance not only the antimicrobial and antioxidant effect, but also the regenerative and protective impact on the skin.

Indeed, snail mucus has attracted considerable attention as a promising source of bioactive compounds with potential pharmaceutical applications. However, to the best of our knowledge, no previous study has investigated the chemical composition and biological properties of mucus derived from the Moroccan endemic snail *O. tingitana*. To address this knowledge gap, the present study aims to evaluate the chemical composition, antibacterial activity, antioxidant capacity, anti-inflammatory properties, and wound-healing potential of *O. tingitana* mucus using *in vitro* and *in vivo* assays. In addition, the study aims to examine these properties under different dietary regimens, in which *O. tingitana* snails are fed a basal diet or diets supplemented with 3% of *Rosmarinus officinalis*, *Origanum compactum*, or *Thymus zygis* subsp. *zygis*, in order to assess the potential influence of diet on mucus bioactivity.

## 2. Results

### 2.1. Chemical Profile of O. tingitana Mucus

Chemical composition of *O. tingitana* mucus, following trimethylsilyl (TMS) derivatization, was determined using the GC-MS method and results are summarized in Table 1. The results revealed that *O. tingitana* mucus had different chemical compositions, including difference in the types of molecules and their diversity based on feed supplementation. Specifically, the largest diversity of components was observed in snail mucus fed with medicinal plants (Supplementary Figures S1–S5).

In this study, GC-MS revealed the detection of 13 different compounds, belonging to phenolic metabolites, fatty-acid related compounds, carbohydrate derivatives, and sterol derivative compounds. The results showed that 9-Octadecenoic acid (oleic acid), TMS derivative is the main compound detected in mucus from snails fed a basic diet (group A), snails fed on flour with *R. officinalis* (group B), and snails fed on flour with *O. compactum* (group C), while salicylic acid, 2TMS derivative was detected as the major compound in mucus from snails fed on flour with *T. zygis* (group D) and wild *O. tingitana* snails (group E). Although these compounds have previously been reported in the mucus of other gastropod species, this study represents the first attempt to characterize the chemical composition of *O. tingitana* mucus.

### 2.2. Antibacterial Activity

The antibacterial activity of *O. tingitana* mucus samples was assessed using agar disc-diffusion and broth microdilution assays. In the disc-diffusion test, all mucus samples produced measurable inhibition zones ranging from 10.31 ± 0.10 to 14.30 ± 0.30 mm depending on the bacterial strain and diet group (Table 2). The positive control (amoxicillin, 10 µg) produced inhibition zones within the expected assay range, confirming the validity of the experimental conditions. Broth microdilution analysis further demonstrated inhibitory activity, with minimum inhibitory concentrations (MICs) ranging from 120 to 240 µg/mL across the tested strains. Corresponding minimum bactericidal concentrations (MBCs) yielded MBC/MIC ratios below 4, indicating predominantly bactericidal behavior under the tested conditions (Table 3). Comparative analysis among diet groups showed that mucus obtained from snails fed with medicinal plants generally exhibited lower MIC values and larger inhibition zones than mucus from the basal-diet (flour) group, suggesting that dietary supplementation may enhance antibacterial performance (*p* < 0.05). This trend was particularly evident against *S. aureus*, where the lowest MIC value (120 µg/mL) was observed in group C (fed with *O. compactum*), compared to 220 µg/mL for the basal diet group (group A), representing nearly a two-fold increase in efficacy. Similarly, against *L. monocytogenes*, MIC values decreased from 180 µg/mL in group A (fed basal diet) to 160 µg/mL in supplemented groups (B, C, and D), accompanied by a marked reduction in MBC values (from 700 TO 500–300 µg/mL), indicating improved bactericidal performance.

### 2.3. Antioxidant Capacity

The antioxidant capacity of *O. tingitana* mucus was significantly influenced by diet, as evidenced by the IC_50_ values (Table 4). Among the tested groups, mucus from snails fed with *O. compactum* (group C) exhibited the strongest antioxidant activity (IC_50_ = 1.183 ± 0.06 mg/mL), followed by those fed with *T. zygis* (group D; IC_50_ = 1.850 ± 0.03 mg/mL) and wild snails (group E; IC_50_ = 3.904 ± 0.08 mg/mL) (*p* < 0.05). In contrast, snails fed with *R. officinalis* (group B) and the control group fed with the basal diet (group A) showed significantly lower antioxidant activity, with IC_50_ values of 6.984 ± 0.04 mg/mL and 28.369 ± 0.04 mg/mL, respectively. As expected, ascorbic acid exhibited the highest antioxidant capacity (IC_50_ = 0.929 ± 0.002 mg/mL). Overall, lower IC_50_ values correspond to higher antioxidant activity, indicating that mucus obtained from snails fed plant-supplemented diets possesses enhanced antioxidant potential.

### 2.4. Wound Healing Effect

**Figure 1:**
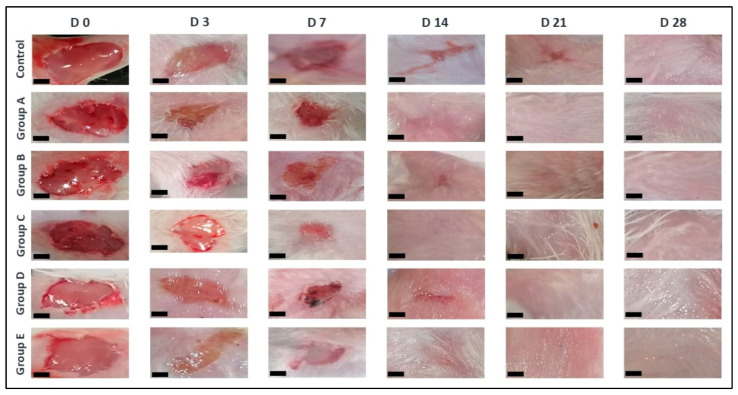
Evolution of wound healing after treatment with different samples of *O. tingitana* snail mucus. D: day. Group A: snails fed on flour only; group B: snails fed on flour supplemented with 3% *R. officinalis*; group C: snails fed on flour supplemented with 3% *O. compactum*; group D: snails fed on flour supplemented with 3% *T. zygis*; group E: wild *O. tingitana*. Scale bar = 0.1 cm.

**Figure 2:**
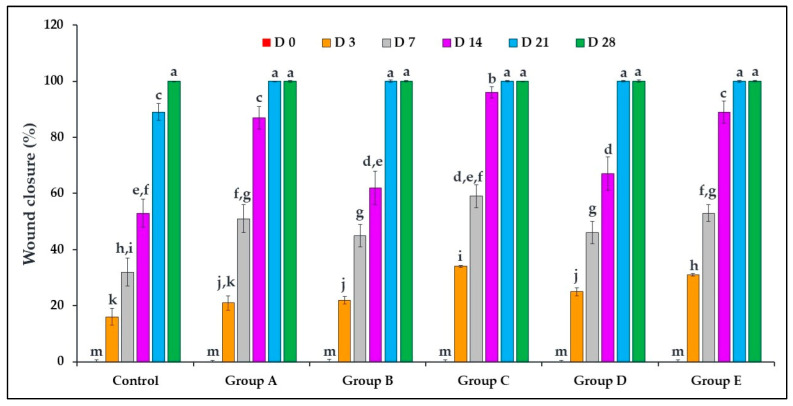
Percentage of wound closure after treatment with *O. tingitana* mucus samples. Group A: snails fed on flour only; group B: snails fed on flour supplemented with 3% *R. officinalis*; group C: snails fed on flour supplemented with 3% *O. compactum*; group D: snails fed on flour supplemented with 3% *T. zygis*; group E: wild *O. tingitana*. Different letters indicate statistically significant differences between groups (ANOVA followed by Fisher’s least significant difference (LSD) test for post hoc comparisons; *p* < 0.05).

In this study, mice were treated with different *O. tingitana* mucus samples, and the development of wounds was closely monitored over time (Figure 1). The visual assessment of wound healing clearly showed a faster closure in all the treated groups compared to the untreated group (control). In addition, the quantitative assessment, based on wound contraction percentage, further confirmed these observations, revealing a significantly accelerated healing rate in mucus-treated groups (*p* < 0.05). Complete wound closure was achieved in less than 21 days in the treated groups, whereas the control group required up to 28 days (Figure 2). Particularly, mucus obtained from snails fed with *O. compactum* (group C) exhibited the most pronounced healing effect, followed by groups E, A, D, and B. The untreated control group showed delayed wound contraction and prolonged healing. The combination of visual and quantitative evidence strongly supports the conclusion that *O. tingitana* mucus significantly enhances wound healing in a diet-dependent manner.

### 2.5. Anti-Inflammatory Effect

The anti-inflammatory effect of snail mucus was assessed by determining the degree of inflammatory cell infiltration in each wound tissue sample. This evaluation was performed through direct observation under a light microscope (Supplementary Images), and results are expressed semi-quantitatively as follows: − (no inflammatory reaction); + (less than 10% inflammatory cells); ++ (10–50% inflammatory cells); +++ (more than 50% inflammatory cells).

Histological examination of wound tissues revealed pronounced inflammatory cell infiltration three days (D_3_) after injury in all groups, with inflammatory cells accounting for more than 50% of the tissue area, mainly due to neutrophil infiltration and disruption of the dermal structure. By day 7 (D_7_), a clear reduction in inflammatory cell infiltration was observed in the group treated with *O. tingitana* mucus. Groups A, C, D, and E correspond to snails fed the basal flour diet, *O. compactum*, *T. zygis*, and wild snails, respectively, showing less than 10% inflammatory cell infiltration, indicating mild inflammation, whereas group B, fed *R. officinalis*, still exhibited moderate inflammatory cell infiltration (10–50%). By day 14 (D_14_), inflammation, as marked by inflammatory cell infiltration, had completely resolved in groups A, C, and E and was reduced to minor levels in groups B and D, while the control group continued to show severe inflammatory cell infiltration. By day 21 (D_21_), the control group still exhibited noticeable inflammatory cell infiltration, whereas all groups treated with *O. tingitana* mucus showed complete resolution of inflammation. Finally, by day 28 (D_28_), full resolution of inflammation was observed across all groups, including the control group (Table 5).

## 3. Discussion

In recent years, snail mucus has drawn a lot of scientific attention because of its rich composition of bioactive substances and prospective uses in various fields, such as medicine, pharmacy, and cosmetics [9,16,24]. This study examined the chemical composition of snail mucus from *O. tingitana* and assessed its antibacterial, antioxidant, wound-healing, and anti-inflammatory properties, with particular focus on the influence of plant-based dietary supplementation.

Chemical composition analysis using GC-MS allowed a tentative identification of 13 different compounds, with the dominance of 9-Octadecenoic acid (oleic acid), TMS derivative in mucus extracted from snails fed a basal diet (group A), snails fed on flour with 3% *R. officinalis* (group B), and snails fed on flour with 3% *O. compactum* (group C), and the dominance of salicylic acid, 2TMS derivative in mucus from snails fed on flour with 3% *T. zygis* (group D) and wild *O. tingitana* snails (group E). These results differ from those reported in the mucus of *Helix aspersa* that showed the high dominance of cyclotrisiloxane, hexamethyl with 47.303% [14]. Similarly, a study carried out by Sallam et al. [25] showed the dominance of Oxime-, methoxy-pheny in the mucus from land snail species, including *Eobania vermiculata*, *Theba pisana*, and *Monacha obstructa*. In addition, EL-Zawawy et al. [15], identified phthalic acid, 7-bromoheptyl ethyl ester as the major compound in the mucus of *E. desertorum*. Nevertheless, several of the compounds identified in the present study have also been detected in the mucus of other gastropods, including *Archachatina marginata* [26] and *Cornu aspersum* [27]. These findings suggest the presence of partially conserved biochemical features across terrestrial snail mucus. Moreover, the results revealed a high diversity in the distribution of chemical components among mucus samples from snails fed a diet supplemented with different plants, indicating that the chemical composition of snail mucus is strongly influenced by dietary supplementation. Indeed, heliciculture under controlled environmental conditions, combined with tailored diets, represents a promising strategy to enrich snails’ mucus with desired bioactive components.

The antibacterial assessment demonstrated that *O. tingitana* mucus exhibits notable inhibitory activity against both Gram-positive and Gram-negative bacteria. Although the antibacterial properties of *O. tingitana* mucus have not previously been reported, comparable inhibitory effects have been described for mucus obtained from other terrestrial snail species, including *Helix aspersa* and related gastropods, supporting the broader antimicrobial potential of snail secretions [28,29,30,31]. The antimicrobial performance of snail mucus is generally attributed to the synergistic action of multiple biochemical components, including antimicrobial peptides, glycoproteins, enzymes, and low-molecular-weight metabolites, rather than to single dominant compounds. Because chemical annotations derived from chromatographic profiling remain tentative without targeted isolation and validation, the present findings should be interpreted as reflecting the overall functional activity of the mucus matrix rather than the effect of specific individual constituents. Nevertheless, previous studies have reported antibacterial properties of several compounds detected in this study including 9-Octadecenoic acid, 5,8,11,14-Eicosatetraenoic acid (arachidonic acid), and salicylic acid [32,33,34]. Notably, mucus obtained from snails receiving diets supplemented with medicinal plants showed improved antibacterial performance compared with mucus from the basal-diet group. This observation suggests that dietary composition may influence physiological metabolism and secretion composition in gastropods, potentially affecting antimicrobial peptide expression, immune-related biochemical pathways, or general metabolic status. Similar diet-associated modulation of bioactive secretions has been proposed in other biological systems, although the precise mechanisms in *O. tingitana* require further targeted investigation.

The imbalance between free radicals and antioxidant defenses within the organism leads to oxidative stress, which contributes to the development of numerous diseases and accelerates aging processes. Consequently, the research for potent antioxidant compounds has attracted increasing attention in recent years. In this context, the present study demonstrated that *O. tingitana* mucus possesses interesting antioxidant activity, as evidenced by its strong DPPH radical scavenging activity. These findings are consistent with previous studies describing antioxidant properties in the mucus of other terrestrial snail species, including *H. aspersa* Muller and *E. desertorum* [35,36]. Importantly, dietary supplementation with medicinal plants significantly enhanced the antioxidant potential of the mucus. This improvement may be attributed to the presence of bioactive compounds tentatively detected by GC-MS, including phenolic derivatives such as salicylic acid derivatives, which are well known for their strong antioxidant properties [37]. In addition, compounds such as 9,10-secocholesta derivatives and unsaturated fatty acids may act synergistically to reinforce the overall antioxidant effect [38]. Snail fed diets enriched with medicinal plants, particularly *O. compactum* (group C) and *T. zygis* (group D) exhibited higher antioxidant activity, suggesting that dietary bioactive compounds can be incorporated into mucus secretions and enhance their radical-scavenging capacity. Overall, these results highlight a strong relationship between diet-modulated chemical composition and antioxidant potential, emphasizing the role of plant-based feeding strategies in heliciculture as a promising approach to enhance the functional bioactivity of snail mucus and its potential therapeutic applications against oxidative damage.

Furthermore, the mucus of *O. tingitana* demonstrated promising wound-healing efficacy along with notable anti-inflammatory properties. Wound healing is a complex and tightly regulated biological process involving sequential but overlapping phases, including inflammation, proliferation, and tissue remodeling, all of which require coordinated cellular and molecular events [34]. Previous studies showed that snail mucus can support key processes essential for tissues repair, such as collagen deposition, angiogenesis, and keratinocyte migration, which collectively contribute to improved wound closure and tissue strength [8,9,10,12,14,39,40]. In the present study, the application of mucus derived from snails fed with medicinal plant-supplemented diets enhances wound closure while simultaneously attenuating the degree of inflammatory cell infiltration, highlighting its dual role in tissue regeneration and immune modulation.

The observed anti-inflammatory effect was characterized by a reduction in the degree of inflammatory cell infiltration, particularly, neutrophils, which are key mediators of early inflammatory response [41]. While inflammation is essential for host defense and tissue repair, excessive or prolonged inflammatory activity can lead to oxidative stress, extracellular matrix degradation, and delayed healing [42]. Therefore, the ability of *O. tingitana* mucus to modulate the inflammatory response suggests a beneficial regulatory effect that supports efficient tissue repair. This rapid attenuation of inflammation may involve reduced neutrophil infiltration and subsequent limitation of reactive oxygen species (ROS) production, thereby minimizing secondary tissue damage [43]. Moreover, antioxidant and anti-inflammatory compounds present in snail mucus may further reduce oxidative stress and limit tissue injury by scavenging reactive oxygen species and modulating inflammatory signaling pathways [44]. Notably, the effective wound healing observed in the group receiving the plant-supplemented diet may be attributed to its richness in antioxidants and bioactive metabolites, suggesting a strong link between diet-induced modulation of mucus composition and biological performance. Overall, these findings highlight a synergistic interplay between the reduction of inflammatory cell infiltration and complete tissue regeneration, as evidenced by improved epidermal restoration and skin appendage formation by day 28. Collectively, the results underscore a strong correlation between the chemical composition of *O. tingitana* mucus and its capacity to modulate key processes involved in wound healing and inflammation control.

In summary, results of this investigation have important implications for the potential applications of *O. tingitana* mucus in pharmaceutical and biomedical fields. Its major biological properties, including antibacterial, anti-inflammatory, wound healing, and antioxidant activity, highlight its effectiveness as a natural bioactive product with potential therapeutic value in treating various diseases. Furthermore, the enhancement of these properties through medicinal plant supplementation of snail feed underscores the promise of dietary strategies in heliciculture to optimize and maximize the bioactive potential of snail mucus.

## 4. Materials and Methods

### 4.1. Snail Animals and Material Plants

A total of 360 one-old day *O. tingitana* snails were used for this study. The snails were randomly separated into four groups of 90 individuals and placed each in a separate clear polystyrene box with perforated tops. Group A was fed a diet consisting only of flour, group B was given flour containing 3% of *R. officinalis* powder, group C was given flour containing 3% of *O. compactum* powder, and group D was given flour containing 3% of *T. zygis* powder (Table 4). All the snail groups were reared for 140 days under carefully regulated conditions with a temperature of 20 °C, humidity of 80%, and an 8/16 h light/dark cycle [18]. Feed was distributed to the snails three times a week in their respective raising boxes.

### 4.2. Preparation of O. tingitana Mucus

The extraction of snail mucus samples from different snail groups was processed using the same standardized extraction protocol following a previously published method [45], with few modifications. To induce the secretion of an adequate amount of mucus, each snail was gently stimulated using a sterile wooden stick, which was carefully rubbed over its muscular foot. Then, the secreted mucus was collected in a sterile container and macerated in sterile distilled water (1:2; *v*/*v*). The samples were then centrifuged at 6000× *g* for 30 min at 4 °C (Eppendorf 5804 R, Hamburg, Germany) to remove cell debris and insoluble particles. The supernatant obtained was collected and stored at −20 °C for no longer than six days prior to analysis. The collected snail mucus samples were divided into 5 groups: group A obtained from snails fed with only flour, group B from snails fed with *R. officinalis*, group C from snails fed with *O. compactum*, group D from snails fed with *T. zygis*, while for comparison, we added group E for mucus obtained from wild *O. tingitana* snails (Table 4).

For analysis, liquid–liquid extraction was performed using hexane as the organic solvent. After vigorous shaking and phase separation, the hexane (Sigma-Aldrich, Darmstadt, Germany) phase was recovered, dried over anhydrous sodium sulfate (Na_2_SO_4_, Sigma-Aldrich, Darmstadt, Germany)), and filtered. The solvent was subsequently removed by gentle evaporation using a rotary evaporator (Büchi, Flawil, Switzerland) at low temperature (≤40 °C) until dryness, yielding a concentrated organic residue. This residue constituted the extract used for chemical characterization and biological investigations. For GC–MS analysis, derivatization was performed according to a recently published protocol with minor modifications [46]. Briefly, 10 mg of the dried extract was dissolved in 200 µL of anhydrous pyridine (Sigma-Aldrich, Darmstadt, Germany), followed by the addition of 100 µL of N,O-bis(trimethylsilyl)trifluoroacetamide (BSTFA) containing 1% trimethylchlorosilane (TMCS) (Sigma-Aldrich, Darmstadt, Germany). The reaction mixture was vortexed and incubated at 70 °C for 30 min to allow trimethylsilyl (TMS) derivatization. After cooling to room temperature, the derivatized sample was directly subjected to GC–MS analysis.

### 4.3. GC-MS Analysis of O. tingitana Mucuss

The analyses were performed by gas chromatography coupled with mass spectrometry (GC-MS) using an Agilent 6890 chromatograph coupled to an Agilent 5973 mass selective detector (Agilent Technologies, Santa Clara, CA, USA) [25]. Chromatographic separation was achieved on an HP-5MS capillary column (5% phenyl polysiloxane, non-polar phase) measuring 30 m × 0.25 mm i.d. and 0.25 µm film thickness. The injection was performed in splitless mode with a volume of 1 µL, and the injector temperature was set at 250 °C. Helium was used as the carrier gas at a constant flow rate of 1.0 mL/min. The oven temperature was programmed at 8 °C/min up to 260 °C, resulting in a total run time of 45 min.

Mass spectrometry was performed using electron impact ionization (EI) at 70 eV in full scan mode, with a mass range of *m*/*z* 50–500. The quadrupole temperature was set at 150 °C and the ion source temperature at 230 °C. The electron multiplier voltage was set at 1100 V. Instrument tuning and calibration were performed using perfluorotributylamine (PFTBA).

Compound identification was based on comparison of the obtained mass spectra with the NIST and Wiley 7n.1 libraries. A similarity index ≥ 85% was used as the minimum criterion of tentative identification. Matches were further verified by manual inspection of characteristic fragment ions and fragmentation patterns.

Linear retention indices (RI) were calculated using a homologous series of n-alkanes (C_7_–C_30_) analyzed under the same conditions. The RIs were determined using the Kovats formula and compared with values reported in the literature and reference databases. Only compounds showing good agreement between mass spectra and retention indices were retained.

Blank analyses, including solvent blank, procedural blanks (all preparation steps without mucus), and empty tubes or vials, were systematically performed. Compounds detected in blanks were considered contaminants and excluded from the results. Only compounds absent from the blanks were considered for further analysis.

Identifications deemed biologically implausible or inconsistent with GC–MS analysis were critically re-evaluated based on spectral quality, retention indices, and available reference data. Compounds that did not meet these criteria were excluded from the final list.

### 4.4. Antibacterial Activity

#### 4.4.1. Bacterial Strains

To conduct antibacterial activity, four bacterial strains, belonging to Gram-negative and Gram-positive bacteria, were selected for this study. The tested bacterial strains include *S. aureus* ATCC 43300, *L. monocytogenes* ATCC 19115, *E. coli* ATCC 35218, and *S.* Typhimurium ATCC 13311. From stock culture, a loopful from each strain was inoculated in Mueller–Hinton Agar (MHA, Biokar, Beauvais, France) and incubated at 37 °C for 24 h. After incubation, a bacterial suspension (0.5 McFarland;10^8^ CFU/mL) was prepared in sterile physiological water (0.9% NaCl) and used for the following experiments.

#### 4.4.2. Disc Diffusion Method

Antibacterial activity of *O. tingitana* mucus groups (A–E) was evaluated using a standardized agar disc-diffusion method with minor modifications of the protocol described by Ed-Dra et al. [47]. The tested bacterial strains were freshly cultured on Mueller–Hinton Agar (MHA, Biokar, Beauvais, France) and adjusted to 0.5 McFarland standard (≈1 × 10^8^ CFU/mL). Sterile cotton swabs were used to uniformly spread 100 µL of each bacterial suspension over the entire surface of MHA plates to obtain a confluent lawn. Sterile 6 mm diameter filter-paper discs were loaded with 10 µL of freshly prepared snail mucus samples (A–E) and allowed to absorb completely under aseptic conditions. The discs were then gently placed on the inoculated agar surface using sterile forceps. Plates were incubated at 37 °C for 24 h under aerobic conditions. Following incubation, antibacterial activity was assessed by measuring the diameter of the inhibition zones in millimeters, including the disc diameter, using a calibrated ruler. Sterile distilled water-loaded discs (10 µL) served as negative controls, while commercial amoxicillin discs (10 µg, Oxoid, Hampshire, UK) were used as positive controls. Each assay was performed in triplicate on independent plates, and results were expressed as mean ± standard deviation.

#### 4.4.3. Microdilution Method

The microdilution method was used to determine the minimum inhibitory and minimum bactericidal concentrations (MIC, MBC) following a previously published method with few modifications [48]. In 96-well microplates, different dilutions of mucus samples were prepared in 50 µL of sterile distilled water. Then, 50 µL of bacterial strains (0.5 McFarland) was added to each well, following the addition of 100 µL of Mueller--Hinton Broth (MHB, Biokar, Beauvais, France). The well containing only snail mucus and MHB was used as the negative control while amoxicillin (0.25–256 ug/mL) was used as the positive control. Afterwards, the microplates were incubated at 37 °C for 24 h. After incubation, bacterial growth was revealed by pink–red coloration after the addition of 40 µL of TTC (2,3,5-triphenyl-tetrazolium chloride, HiMedia, Mumbai, India) with a concentration of 0.2 g/mL, and incubation for 30 min at 37 °C. MIC was determined as the lower snail mucus concentration at which no bacterial growth was observed.

To determine MBC, 5 µL from each well that did not exhibit bacterial growth was inoculated on MHA and incubated at 37 °C for 24 h. The MBC was determined as the lower snail mucus concentration that did not show colony growth on MHA. Moreover, the ratio MBC/MIC was used to determine the bactericidal or bacteriostatic effect of snail mucus. In fact, the sample presents a bactericidal effect if the ratio MBC/MIC is equal to or lower than 4; if not, it presents a bacteriostatic effect [18].

The CMI and CMB concentrations are expressed in micrograms per milliliter (µg/mL). The values reported correspond exclusively to the concentrations prepared from the concentrated organic extract (considered as pure product) obtained from snail mucus according to the protocol described in Section 4.2.

### 4.5. Antioxidant Capacity

The DPPH radical experiment was carried out to examine the capacity of *O. tingitana* mucus to scavenge free radicals, following a previously published protocol with few modifications [48]. A series of 2-fold dilutions of snail mucus were prepared in distilled water. Then, 0.5 mL of each dilution was mixed with 1.5 mL of DPPH methanol solution (0.025 g/L) (Sigma-Aldrich, Darmstadt, Germany) and incubated for 30 min at room temperature under light protection. A tube containing DPPH and methanol (without mucus samples) was used as the negative control. After incubation, the absorbance of both negative control and samples was measured at 517 nm using a Shimadzu UV-1601 spectrophotometer (Nakagyo-ku, Japan), and the percentage of inhibition was calculated using the following formula:

$$I\;\left(\%\right)=\frac{Abs\;control-Abs\;sample}{Abs\;control}\times 100$$

Ascorbic acid was used as the positive control of this assay [48], and IC_50_ related to the concentration of snail mucus providing 50% inhibition was determined and presented as the average ± standard deviation (SD) of three replicates.

### 4.6. Wound-Healing Assessment

The *in vivo* assays were conducted using adult male BALB/c mice (8–10 weeks old), weighting between 20 and 30 g. The mice were sourced from the animal facility of the Department of Biology at Moulay Ismail University, Faculty of Sciences, Meknes, Morocco. This facility is designed and managed in strict compliance with national regulations and ethical principles relating to the use of animals for scientific purposes. To prepare animals for *in vivo* experiments, mice were housed individually in suitable cages under controlled environmental conditions (temperature of 22 °C, 12-light/dark cycle), with ad libitum access to water and food.

To assess the *in vivo* wound-healing properties of *O. tingitana* mucus, mice were divided into six experimental groups (each group was treated with a specific group of snail mucus), with five animals per group (n = 5). Each experiment was repeated three independent times under identical conditions, resulting in a total of 90 animals (Table 6). No exclusions were made, and all animals were included in the final analysis.

To achieve the objectives of this trial, standardized 0.5 cm^2^ square lesions were made on the mice’s backs after anesthesia [49]. Then the mice were randomly divided into six groups of five individuals, and each group was repeated three times under identical conditions. Each group received daily topical applications of mucus from snails fed with specific diets: group A was treated with snail mucus fed with a basic flour diet, group B was treated with snail mucus fed with *R. officinalis*, group C was treated with snail mucus fed with *O. compactum*, group D was treated with snail mucus fed with *T. zygis*, and group E was treated with mucus from wild snails. In addition, a group without treatment was used as the control. The treatment was applied daily until the wound closed. The mucus’s bioactive effectiveness was evaluated by calculating the percentage of wound closure using the following formula [14]:

$$Wound\;closure\;(\%)=\frac{Wound\;size\;at\;T0-Wound\;size\;at\;Tf}{Size\;of\;wound\;at\;T0}\times 100$$

where T_0_ denotes the initial day (D_0_) of the experiment, and T_f_ denotes the day of wound size assessment (D_3_, D_7_, D_14_, D_21_, D_28_).

All animal experiments were conducted in accordance with international guidelines on animal welfare and were approved by the institutional ethical committee for the care and use of laboratory animals at the Faculty of Sciences of Meknes, Moulay Ismail University, Morocco (approval code: 4A-87; approval on 14 July 2025).

### 4.7. Anti-Inflammatory Activity

The experimental protocol and animal allocation described in Section 4.6 were used to evaluate the anti-inflammatory activity of *O. tingitana* mucus. This activity was assessed semi-quantitatively based on the degree of inflammatory cell infiltration observed in each tissue sample under a light microscope.

The inflammation was naturally induced by the skin lesion itself, and no additional inflammatory agent was administered. On days 3, 7, 14, 21, and 28 following the induction of a skin lesion, one animal from each group (three replicates per group) was humanely euthanized for tissue collection. Euthanasia was performed by gradual carbon dioxide (CO_2_) inhalation in a well-ventilated chamber. Animals were exposed to CO_2_ until loss of consciousness and cessation of respiratory movements occurred [50]. Death was confirmed by the absence of reflexes and breathing before the collection of tissue samples. Immediately after euthanasia, the dorsal wound area along with the surrounding skin was carefully excised under sterile conditions for subsequent histological analyses. Three biological repetitions were performed for each treated group (A–E) and the control. All procedures were conducted in accordance with internationally accepted ethical guidelines for the care and use of laboratory animals, with measures implemented to minimize animal suffering [50].

Afterwards, tissue samples were embedded in paraffin and preserved in formalin according to previously published methods [51]. To examine the degree of inflammatory cell infiltration, Hematoxylin–eosin staining was applied to serial 5 µm slices. Using histological analysis, the degree of inflammation was classified as follows: − (no inflammatory reaction), + (inflammatory cells < 10% of the cellular population in the wound area), ++ (inflammatory cells 10–50%), and +++ (inflammatory cells > 50%).

### 4.8. Ethical Approval

All animal experiments were conducted in accordance with internationally recognized ethical guidelines, including the European Directive 86/609/EEC and the Guide for the Care and Use of Laboratory Animals published by the National Institutes of Health (NIH Publication No. 85-23, revised 1985). The study protocol was reviewed and approved by the Institutional Ethics Committee for the Care and Use of Laboratory Animals at the Faculty of Science of Meknes, Moulay Ismail University (approval code: 4A-87; approval on 14 July 2025). All necessary measures were taken to minimize animal suffering and to ensure full compliance with the principles of humane care and use of laboratory animals.

### 4.9. Data Analysis

All measurements were performed at least in triplicates and results were expressed as mean values ± standard deviation (SD). Differences between groups were assessed using one-way ANOVA followed by Tukey’s post hoc test for multiple comparisons when data met parametric assumptions.

Wound closure percentages were measured longitudinally in the same animals at multiple time points (D_3_, D_7_, D_14_, D_21_, and D_28_). The data were analyzed using two-way ANOVA followed by Fisher’s least significant difference (LSD) test for post hoc comparisons. The assumptions of normality and homogeneity were evaluated prior to analysis. The degree of inflammatory cell infiltration was assessed semi-quantitatively as follows: − (no inflammatory reaction), + (<10% inflammatory cells), ++ (10–50% inflammatory cells), and +++ (>50% inflammatory cells). These scores were converted into ordinal values (0–3), and differences between groups were analyzed using the Kruskal–Wallis test, followed by Dunn’s multiple comparison test when a significant difference was detected. Data analysis was performed using GraphPad Prism version 8 (GraphPad, San Diego, CA, USA) and Microsoft Excel (New York, NY, USA) software. A *p* < 0.05 was considered for statistically significant difference.

## 5. Conclusions

To the best of our knowledge, this study provides the first integrated characterization of mucus from the Moroccan terrestrial snail *O. tingitana*, combining chemical profiling with the evaluation of its antibacterial activity, antioxidant capacity, wound-healing potential, and anti-inflammatory effects. The results demonstrate that *O. tingitana* mucus exhibits notable biological activities and that dietary supplementation with medicinal plants can significantly influence its biochemical composition and biological properties. These findings support the study objectives by highlighting the potential of diet-based strategies in heliciculture to modulate the functional properties of snail mucus.

However, several limitations should be acknowledged. Although GC-MS analysis provided a preliminary overview of the chemical composition, the identified compounds remain tentative and require further isolation and structural confirmation. In addition, the use of crude mucus limits the direct attribution of the observed biological activities to specific bioactive molecules. Therefore, the relationship between the detected chemical compounds and the measured biological effects remains to be clarified. Moreover, anti-inflammatory potential should be further investigated by incorporating biochemical inflammatory markers alongside histological tissue assessment, as well as by including standard reference drugs for comparative evaluation of activity.

Future research should therefore focus on targeted biochemical fractionation and purification of mucus components to identify the active compounds responsible for the observed biological effects. Moreover, controlled metabolomic investigations are needed to better understand how heliciculture diet influences mucus composition and functionality. Additional studies should also include expanded antimicrobial testing against clinically relevant resistant pathogens and biofilm models, as well as comprehensive toxicological and dermatological safety assessments. Overall, the present findings highlight the potential of controlled dietary strategies in heliciculture to optimize the functional quality of snail mucus for future biomedical, dermatological, or biomaterial development.

## Figures and Tables

**Table 1:** Comparative chemical composition of *O. tingitana* mucus determined by GC–MS in experimental diet groups and wild snails.

No.	Derivatized Compounds	Formula	Native Compounds	Formula	RT (Min)	RI (Exp)	RI (Lit)	Main Ions (*m*/*z*)	Match (%)	MSI	Area (%)				
											A	B	C	D	E
1	Methyl 4-methoxymandelate, TMS	C_13_H_20_O_4_Si	4-Methoxymandelic acid	C_9_H_10_O_4_	13.22	1542	1550	73, 147, 179, 267	89	2	9.55	-	-	-	3.72
2	Salicylic acid, 2TMS	C_13_H_22_O_3_Si_2_	Salicylic acid	C_7_H_6_O_3_	15.79	1648	1655	73, 147, 267	94	2	6.24	8.6	6.02	19.04	8.64
3	9-Octadecenoic acid (oleic acid), TMS	C_21_H_42_O_2_Si	9-Octadecenoic acid	C_18_H_34_O_2_	18.63	2145	2140	73, 129, 339	96	2	12.89	13.06	15.11	9.71	-
4	2-Methoxy-1,3-dioxolane	C_4_H_8_O_3_	2-Methoxy-1,3-dioxolane	C_4_H_8_O_3_	23.02	1032	1035	45, 75, 103	85	3	-	3.03	-	2.08	-
5	2-deoxy-inosose, O-methyl oxime, TMS	C_19_H_49_NO_5_Si_4_	2-Desoxy-inosose	C_6_H_10_O_5_	23.80	1885	1890	73, 147, 204	87	3	-	-	-	1.83	-
6	5,8,11,14-Eicosatetraynoic acid, TBDMS	C_26_H_38_O_2_Si	5,8,11,14-Eicosatetraynoic acid	C_20_H_24_O_2_	24.51	2440	2450	73, 147, 391	90	2	-	2.70	-	-	2.07
7	2,5-Dihydroxyacetophenone, 2TMS	C_14_H_24_O_3_Si_2_	2,5-Dihydroxyacetophenone	C_8_H_8_O_3_	25.47	1815	1820	73, 147, 281	92	3	-	1.80	-	-	-
8	5,8,11,14-Eicosatetraenoic acid (Arachidonic acid), TMS	C_23_H_32_O_2_Si	5,8,11,14-Eicosatetraenoic acid	C_20_H_32_O_2_	25.61	2135	2140	73, 304, 319	95	2	-	2.70	-	4.04	3.75
9	N-Acetyl-D-glucosamine, methyl glycoside, TMS, cyclic methyl boronate	C_13_H_26_BNO_6_Si	N-Acetyl-D-glucosamine	C_8_H_15_NO_6_	26.40	1952	1950	73, 147, 204	88	2	-	-	-	1.13	-
10	3-Aminobenzoic acid,2TMS derivative	C_13_H_23_NO_2_Si_2_	3-Aminobenzoic acid	C_7_H_7_NO_2_	26.88	1720	1725	73, 147, 266	91	3	-	-	-	-	1.19
11	10,12-Docosadiynedioic acid, 2TMS	C_28_H_50_O_4_Si_2_	10,12-Docosadiynedioic acid	C_22_H_34_O_4_	34.91	2755	2750	73, 147, 433	89	2	-	1.41	-	-	-
12	Catechol, 2TBDMS	C_18_H_34_O_2_Si_2_	Catechol	C_6_H_6_O_2_	37.82	1902	1905	73, 179, 295	93	3	-	-	-	-	1.79
13	9,10-Secocholesta-5,7,10(19)-triene-3,24,25-triol,(3α,5Z,7E), TMS	C_36_H_71_O_3_Si_3_	9,10-Secocholesta-5,7,10(19)-triene-3,24,25-triol,(3α,5Z,7E)-	C_27_H_44_O_3_	39.0	2825	2820	73, 129, 147, 207, 255, 357, 445	85	3	-	-	1.06	-	-

(-): Not detected; RT (min): retention time in minutes; RI (exp): experimental retention index; RI (lit): literature retention index; *m*/*z*: mass-to-charge ratio of main fragment ions; Match (%): spectral similarity with library data; MSI: metabolite identification score; (A): snails group fed on flour only, (B): group fed on flour with 3% *R. officinalis*, (C): group fed on flour with 3% *O. compactum*, (D): group fed on flour with *T. zygis*, (E): wild *O. tingitana*.

**Table 2:** Antibacterial activity of *O. tingitana* mucus against pathogenic bacteria, expressed as inhibition zone diameter (mm).

Snail Mucus Groups	*E. coli*	*S.* Typhimurium	*S. aureus*	*L. monocytogenes*
A	11.13 ± 0.23 ^A^	10.76 ± 0.20 ^A^	10.31 ± 0.10 ^A^	11.16 ± 0.15 ^A^
B	13.16 ± 0.28 ^B^	11.16 ± 0.15 ^A^	11.23 ± 0.20 ^B^	13.26 ± 0.23 ^B^
C	14.30 ± 0.30 ^C^	13.20 ± 0.17 ^C^	12.26 ± 0.25 ^C^	14.20 ± 0.20 ^C^
D	14.13 ± 0.11 ^C^	11.43 ± 0.51 ^A^	12.33 ± 0.28 ^C^	12.13 ± 0.15 ^D^
E	13.00 ± 0.20 ^B^	11.15 ± 0.21 ^A^	13.40 ± 0.34 ^D^	12.16 ± 0.28 ^D^
Amoxicillin	13.20 ± 0.26 ^B^	7.30 ± 0.26 ^B^	11.26 ± 0.25 ^B^	10.16 ± 0.15 ^E^
Sterile distilled water	6.00 ± 0.00 ^D^	6.00 ± 0.00 ^D^	6.00 ± 0.00 ^E^	6.00 ± 0.00 ^F^

(A) snails group fed on flour only, (B) group fed on flour with 3% *R. officinalis*, (C) group fed on flour with 3% *O. compactum*, (D) group fed on flour with *T. zygis*, (E) wild *O. tingitana*; within each column, values followed by the same letter are not significantly different according to one-way ANOVA followed by Tukey’s post hoc test (*p* < 0.05).

**Table 3:** Minimum inhibitory concentration and minimum bactericidal concentration (µg/mL) of *O. tingitana* mucus.

Bacteria		Snail Mucus Groups					
		A	B	C	D	E	AMX
*S. aureus*	MIC	220	140	120	140	180	64
	MBC	500	300	300	300	300	128
	MBC/MIC	2.27	2.14	2.5	2.14	1.66	2
	Effect	Bactericidal	Bactericidal	Bactericidal	Bactericidal	Bactericidal	Bactericidal
*L. monocytogenes*	MIC	180	160	160	160	180	8
	MBC	700	600	500	500	300	16
	MBC/MIC	3.88	3.75	3.12	3.12	1.66	2
	Effect	Bactericidal	Bactericidal	Bactericidal	Bactericidal	Bactericidal	Bactericidal
*S.* Typhimurium	MIC	220	180	220	220	240	64
	MBC	700	500	700	600	600	128
	MBC/MIC	3.18	2.78	3.18	2.73	2.5	2
	Effect	Bactericidal	Bactericidal	Bactericidal	Bactericidal	Bactericidal	Bactericidal
*E. coli*	MIC	240	220	220	220	240	32
	MBC	700	700	700	600	600	64
	MBC/MIC	2.92	3.18	3.18	2.73	2.5	2
	Effect	Bactericidal	Bactericidal	Bactericidal	Bactericidal	Bactericidal	Bactericidal

(A): snails group fed on flour only, (B): group fed on flour with 3% *R. officinalis*, (C): group fed on flour with 3% *O. compactum*, (D): group fed on flour with *T. zygis*, (E): wild *O. tingitana*, (AMX): amoxicillin used as positive control.

**Table 4:** Antioxidant activity of *O. tingitana* snail mucus, expressed as IC_50_ (mg/mL).

Snail Mucus Groups	Diets	IC_50_ (mg/mL)
A	Flour	28.369 ± 0.04 ^A^
B	Flour + 3% *R. officinalis*	6.984 ± 0.04 ^B^
C	Flour + 3% *O. compactum*	1.183 ± 0.06 ^C^
D	Flour + 3% *T. zygis*	1.850 ± 0.03 ^D^
E	Wild snails	3.904 ± 0.08 ^E^
Ascorbic acid	-	0.929 ± 0.002 ^F^

(A) snails group fed on flour only, (B) group fed on flour with 3% *R. officinalis*, (C) group fed on flour with 3% *O. compactum*, (D) group fed on flour with *T. zygis*, (E) wild *O. tingitana*. Different letters indicate statistically significant differences according to one-way ANOVA followed by Tukey’s post hoc (*p* < 0.05).

**Table 5:** The degree of inflammatory cell infiltration measured semi-quantitatively after histological observation of mice skin tissues treated with different *O. tingitana* mucus groups.

	D3	D7	D14	D21	D28
Control	+++	+++	+++	++	-
Group A	+++	+	-	-	-
Group B	+++	++	+	-	-
Group C	+++	+	-	-	-
Group D	+++	+	+	-	-
Group E	+++	+	-	-	-

Group A: snails fed on flour only; group B: snails fed on flour supplemented with 3% *R. officinalis*; group C: snails fed on flour supplemented with 3% *O. compactum*; group D: snails fed on flour supplemented with 3% *T. zygis*; group E: wild *O. tingitana*. Inflammatory response was semi-quantitatively scored as follows: - (no inflammatory reaction); + (less than 10% inflammatory cells); ++ (inflammatory cells accounting for between 10% and 50%); and +++ (inflammatory cells accounting for more than 50%).

**Table 6:** Distribution of animals (mice) across experimental groups in the wound-healing assay.

Experimental Groups	Treatments	Animals/Group	Total Number (3 Repetitions)
Group A	Mucus from snails fed with basic flour diet	5	15
Group B	Mucus from snails fed with *R. officinalis*	5	15
Group C	Mucus from snails fed with *O. compactum*	5	15
Group D	Mucus from snails fed with *T. zygis*	5	15
Group E	Mucus from wild snails	5	15
Control	No treatment	5	15
Total	-	30 animals	90 animals

## Data Availability

The original contributions presented in the study are included in the article/Supplementary Material; further inquiries can be directed to the corresponding authors.

## Supplementary Materials

The following supporting information can be downloaded at https://www.mdpi.com/article/10.3390/molecules31091499/s1.
